# Semi-Siamese U-Net for separation of lung and heart bioimpedance images: A simulation study of thorax EIT

**DOI:** 10.1371/journal.pone.0246071

**Published:** 2021-02-02

**Authors:** Yen-Fen Ko, Kuo-Sheng Cheng

**Affiliations:** Department of Biomedical Engineering, National Cheng Kung University, Tainan, Taiwan; University of Craiova, ROMANIA

## Abstract

Electrical impedance tomography (EIT) is widely used for bedside monitoring of lung ventilation status. Its goal is to reflect the internal conductivity changes and estimate the electrical properties of the tissues in the thorax. However, poor spatial resolution affects EIT image reconstruction to the extent that the heart and lung-related impedance images are barely distinguishable. Several studies have attempted to tackle this problem, and approaches based on decomposition of EIT images using linear transformations have been developed, and recently, U-Net has become a prominent architecture for semantic segmentation. In this paper, we propose a novel semi-Siamese U-Net specifically tailored for EIT application. It is based on the state-of-the-art U-Net, whose structure is modified and extended, forming shared encoder with parallel decoders and has multi-task weighted losses added to adapt to the individual separation tasks. The trained semi-Siamese U-Net model was evaluated with a test dataset, and the results were compared with those of the classical U-Net in terms of Dice similarity coefficient and mean absolute error. Results showed that compared with the classical U-Net, semi-Siamese U-Net exhibited performance improvements of 11.37% and 3.2% in Dice similarity coefficient, and 3.16% and 5.54% in mean absolute error, in terms of heart and lung-impedance image separation, respectively.

## Introduction

### Lung EIT

Lung electrical impedance tomography (EIT) is a promising imaging tool for real-time and non-invasive monitoring of ventilation distribution at bedside [1]. Lung EIT and its clinical applications have evolved over time. Although EIT has limitations with respect to local resolutions, it is sufficient for responding to important clinical therapeutic questions; for example, an EIT-guiding tool is used to optimize positive end-expiratory pressure (PEEP) for acute lung injury (ALI) or acute respiratory distress syndrome (ARDS) patients. Currently, although several tomographic modalities can facilitate higher resolution imaging techniques, EIT provides regional information about the distribution of ventilation as well as changes in end-expiratory lung volume that neither computed tomography (CT) nor functional magnetic resonance imaging (fMRI) methods can provide [2, 3]. EIT has become an increasingly important supplementary imaging technique because of its unique features: the reconstructed images contain new and different information, such as electrical tissue properties and its conductivity distribution in space; moreover, EIT can be applied for continuous bedside monitoring, which only requires portable devices, thus, the patients are not exposed to ionizing radiation. Therefore, these features justify the clinical application of EIT in the ICU wherein it has proven to be the only method that can directly reveal whether collapsed lung regions can be opened by a recruitment maneuver.

Over the past decade, EIT has been applied in clinical research, and numerous EIT-based investigations have been published regarding strategies to optimize alveolar recruitment, maintain an open lung, and avoid pulmonary overdistension [4–6]. Most of the studies on lung EIT focused on ventilation-induced changes, quantification of regional ventilation distribution, and ventilatory status. However, as the technique does not provide sufficient heart-related information, its clinical applicability is limited.

### Lung EIT limitations

The major limitation of the EIT system is that EIT image reconstruction suffers from low spatial resolution in the center region. Tissue characteristic of the center region cannot be determined accurately because the heart is surrounded by the lung, which has relatively lower conductivity, and its location is relatively far for electrodes, causing poor sensitivity to imaging. Therefore, the main challenge is to obtain a higher-quality reconstructed image of the center region to acquire the associated heart-related information. However, the unique advantage of EIT measurement is that it involves not only ventilation distribution changes but also the cardiac-induced related information in EIT images. In other words, if EIT measurement is performed, the reconstructed images obtained depend on conductivity changes in the thorax and present both respiratory and cardiac-related information [7].

Currently, five methods have been proposed to separate the cardiac information from lung respiratory-related information in EIT images: apnea or breath hold, injecting a contrast agent [8], electrocardiography gating [9–12], and Fourier-spectrum-based [13–16] and principal-component-analysis-based [17–19] approaches.

The reported studies have a common problem: there is no consensus standard for validating information on the cardiovascular and respiratory systems status because these approaches were directly implemented based on real EIT data [19]. Another reason for this problem is that other tomographic modalities are not suited for continuous regional lung monitoring at bedside.

Hence, the aim of this study is to establish a finite element method (FEM)-based model of human thorax, which includes the arrangement of low-conductivity lungs on both sides and high-conductivity heart in the middle to simulate the thorax activity in EIT.

### EIT imaging: A nonlinear and ill-posed problem

EIT reconstructed result indicates the internal distribution of conductivity in space by gathering the voltages at the boundary between electrodes and further solving its inverse problem. In other words, EIT imaging is a nonlinear and severely ill-posed inverse problem [20]. In fact, previously proposed methods that assume a linear solution for separation are impractical in real situations to solve the nonlinear problem because the conductivity distribution of the thorax is complicated.

Recently, because of its great advantages in nonlinear problem modeling and feature representation, deep learning has become increasingly popular for solving complex problems [21].

Among the AI-based imaging methods used in EIT, several artificial neural networks (ANNs) algorithms have been investigated, in which supervised learning is utilized to solve the EIT inverse problem. After training, the model can directly determine the conductive distribution from boundary data in a few seconds [22]. These studies have performed bioimpedance reconstruction from real biomedical data. Although ANN is used as an EIT inverse solver for nonlinear reconstruction, it is very sensitive to boundary mismatch and other artifacts in the measured data [23].

Moreover, ANNs are used to enhance the quality of EIT images and reduce the effects of noise and modeling errors after applying a reconstruction algorithm [23]. ANN can be used as a robust postprocessor for reconstructed images. The convolutional neural network-based method for post-processing has subsequently been applied for EIT imaging [24].

It has been shown that AI-based methods can potentially provide an adequate solution for complicated conductivity distribution in a few seconds and are suitable for real-time monitoring.

### U-Net for biomedical image segmentation

Recently, a number of revolutionizing studies for medical image segmentation using deep neural networks have been reported. U-Net has been the most prominent and popular deep-learning-network architecture used in the medical imaging field since it was proposed by Ronneberger et al. [25]. It has been widely used for segmenting medical images because it can be trained from a few images and outperforms other convolutional network methods [26]. There are several applications of U-Net in biomedical image segmentation, such as brain magnetic resonance imaging segmentation [27] and extraction of the liver-lesion images from the CT images [28]. Variants of the U-Net have also been applied for medical image reconstruction; the deep D-bar method is one of the variants that has been developed for enhancing EIT imaging [24]. In the field of medical imaging, U-Net has been used as a powerful model to segment biomedical images. This is similar to our separation application.

However, the separation of lung-related and cardiac impedance changes in EIT images based on the U-Net model has not been studied yet. Moreover, previous studies on separation of bioimpedance images were carried out directly using the real EIT image sequences, and their practical applicability was poor [19] because the separation result would lose some of the ventilation-related information [19, 29]; moreover, the separated image could not reflect the sudden signal changes immediately. Furthermore, those methods were based on the analysis of linear combinations to recompose the lung-related and cardiac images [18]; however, EIT imaging is a markedly nonlinear and ill-posed problem [30]. The variational autoencoder is used to learn solving ill-posed nonlinear inverse problems of EIT [31], which is the well-known encoder-decoder architecture learning technique used in this study.

In this study, a novel method is proposed to separate the lung-related and cardiac impedance changes directly from EIT reconstructed images and enhance the quality of EIT image reconstruction in the center region.

Firstly, we propose some modifications and extensions based on the state-of-the-art U-Net model to tailor for the separation application in EIT.

Secondly, the biomimetics approach is exploited to simulate thorax activity in EIT to validate the separation performance. An FE modeling phantom is established to generate the dataset for training the separation model. The image reconstruction is performed in two dimensions.

The rest of the paper is organized as follows. In sections **Proposed architecture** and **Multi-weighted loss**, we describe the tailored separation architecture “semi-Siamese U-Net,” which exploits the advantages of classical U-Net to modify and extend. The implementation of the FEM-based phantom for preparing the training datasets and the experimental procedure is described in sections **Dataset preparation**, **Training of semi-Siamese U-Net** and **Experiments**. The corresponding separation results of lungs and heart are presented in **Results** section.

## Proposed architecture

The proposed architecture is based on the U-Net architecture, which comprises the state-of-the-art convolutional neural network for medical image segmentation with only a few labelled datasets [25]. This breakthrough architecture has become prominent in the field of medical image segmentation.

The features of the U-Net architecture are applicable to our study, where the aim is to segregate the heart and lung regions in the mixed reconstructed image and decompose individual conductivity distributions. Thus, the output will be two distinguishable impedance images. For this purpose, modifications have been made to the U-Net architecture, tailored for separation application in EIT.

**Fig 1:**
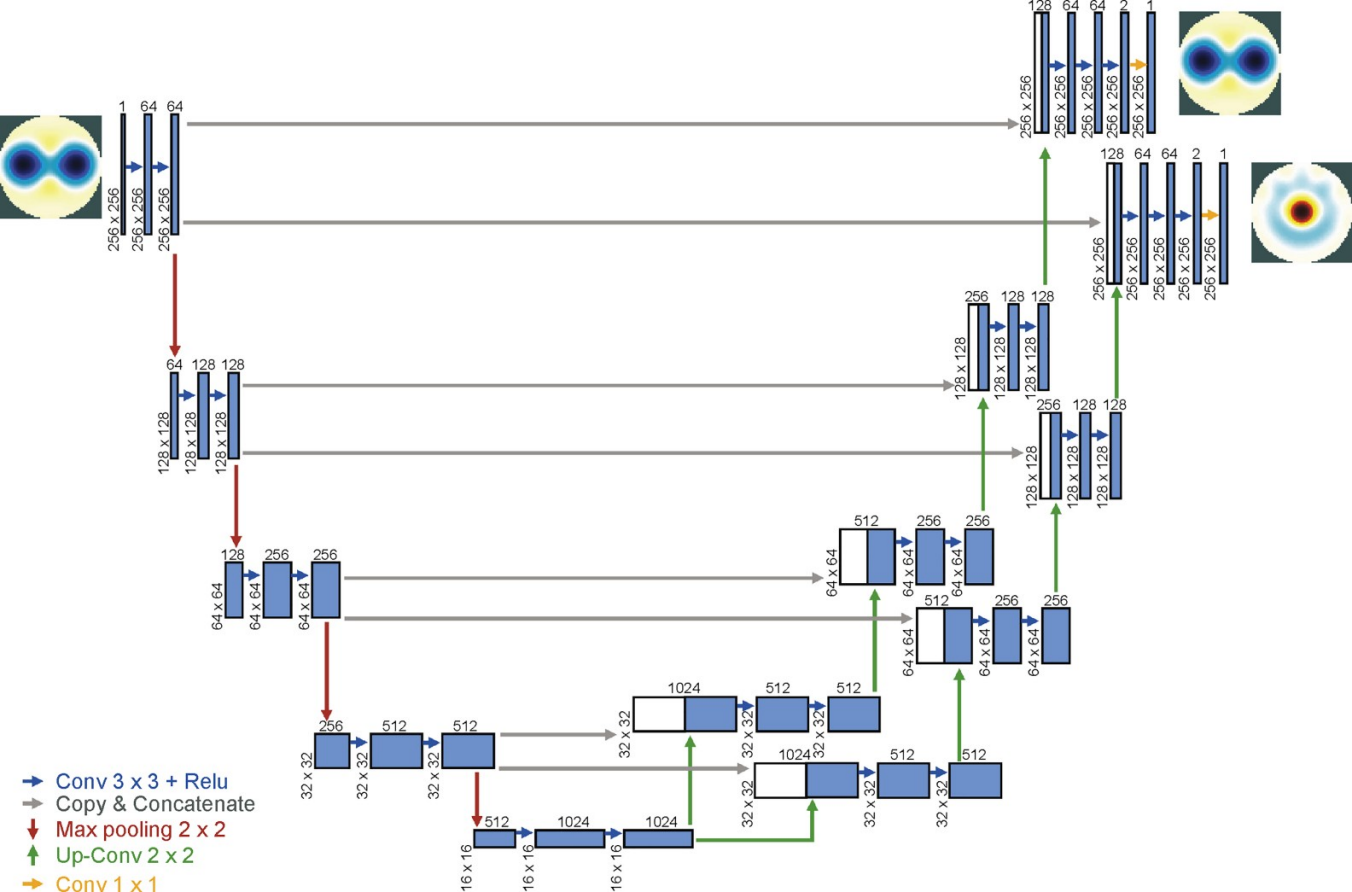
Semi-Siamese U-Net architecture. An illustration of encoder-parallel decoders-based U-Net architecture for EIT co-separation tasks.

In the study, we modified and extended the U-Net architecture, adding a branch from the end of the contracting path to perform the multi-task deep learning for image separation. The novel architecture consists of a contracting path and two parallel expanding paths, to capture the mixed context and precisely localize the individual parts respectively. The two separation tasks were simultaneously process specialized and learned on the expanding paths, which share the parameters and weights on the contracting path in the sharing parameter manner, which works similar to that in the Siamese network concept. Hence, the proposed architecture combines the U-Net and Siamese concept, where the weights are shared in the upper U-Net. We have named this novel architecture “semi-Siamese U-Net,” as shown in Fig 1, which works via joint learning and considers the multiple weighted loss to learn specifically for separating the lung and heart images. The multiple weighted loss is described in the following section.

The segmentation mapping is represented as a deep convolutional neural network that takes the mixed EIT image as the input and outputs the individual parts, i.e., lung-related as well as cardiac images.

Additionally, the dropout layers were performed at the end of the contracting and bottom layers of U-Net to prevent overfitting; the dropout rate is 0.5.

We present the diagram of the proposed semi-Siamese model in Fig 1, and the architectural details are described in Table 1.

**Table 1:** Semi-Siamese U-Net architecture details.

Contracting Path			Expanding Paths		
			Path1 and Path2		
Block	Layer	Filter	Block	Layer	Filter
Block1	Conv2D(3,3)	64	Block 6	Conv2D(2,2)	512
	Conv2D(3,3)	64		Conv2D(3,3)	512
	MaxPooling2D(2,2)			Conv2D(3,3)	512
	Concatenate Block9				
Block2	Conv2D(3,3)	128	Block 7	Conv2D(2,2)	256
	Conv2D(3,3)	128		Conv2D(3,3)	256
	MaxPooling2D(2,2)			Conv2D(3,3)	256
	Concatenate Block8				
Block3	Conv2D(3,3)	256	Block8	Conv2D(2,2)	128
	Conv2D(3,3)	256		Conv2D(3,3)	128
	MaxPooling2D(2,2)			Conv2D(3,3)	128
	Concatenate Bolck7				
Block4	Conv2D(3,3)	512	Block9	Conv2D(2,2)	64
	Conv2D(3,3)	512		Conv2D(3,3)	64
	Dropout			Conv2D(3,3)	64
	MaxPooling2D(2,2)			Conv2D(3,3)	2
	Concatenate Block6				
Block5	Conv2D(3,3)	1024		Conv2D(1,1)	1
	Conv2D(3,3)	1024			
	Dropout				

**Fig 2:**
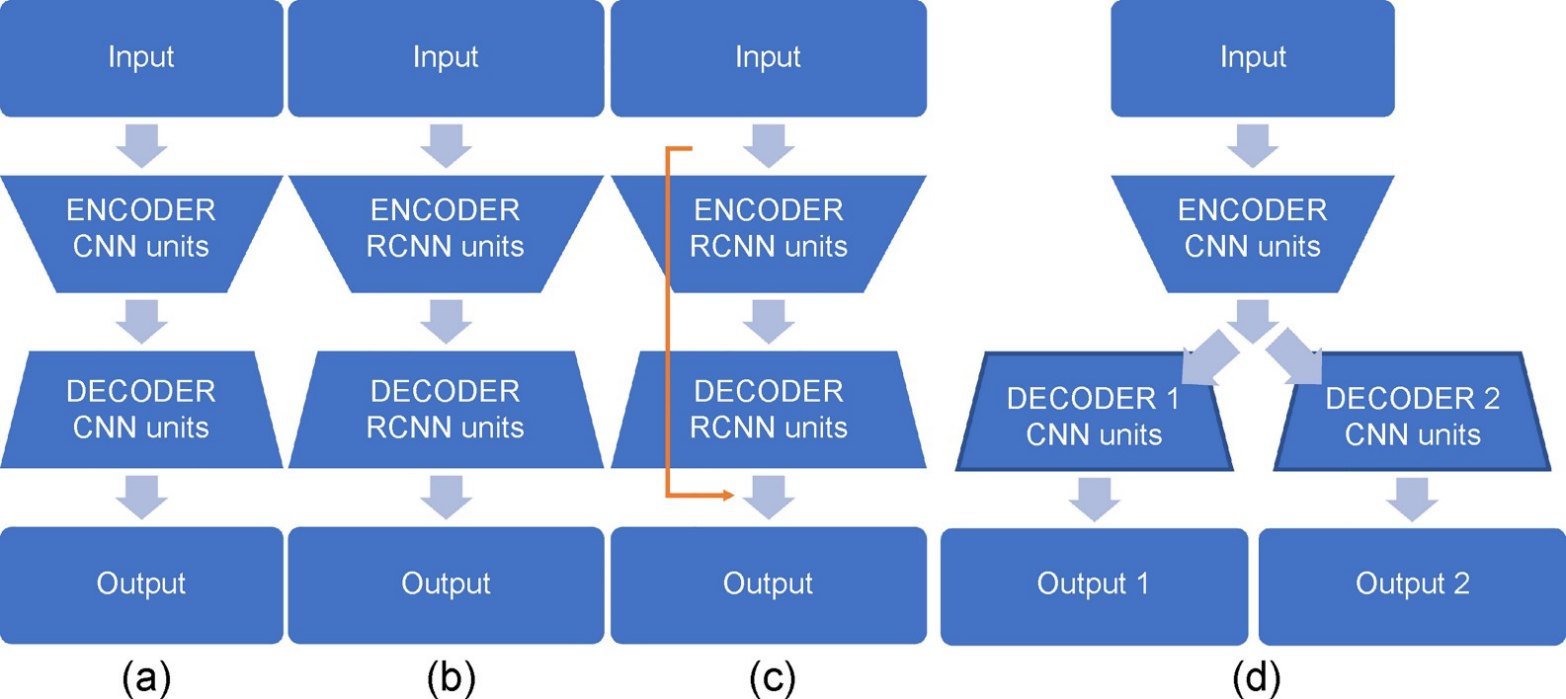
Different variants of U-Net structure and backbone (convolutional and recurrent convolutional units). (a) classical U-Net uses convolutional encoding and decoding unit, (b) RU-Net uses encoder-decoder structure with recurrent convolutional units, (c) R2UNet combines recurrent convolutional units and residual connections (yellow line) form residual RCNN network, and (d) proposed semi-Siamese U-Net which uses encoder-parallel decoders structure with convolutional units.

Detailed comparison of different variants of U-Net and proposed semi-Siamese U-Net model is shown in Fig 2. The classical U-Net, as shown in Fig 2(A), consists of an encoder-decoder structure, along with forward convolutional units. V-Net is an approach based on U-Net to 3D image segmentation using volumetric, fully convolutional neural networks [32]. Based on classical U-Net with modification of backbone as shown in Fig 2(B), RU-Net replaces backbone to recurrent convolution units (RCNN). Besides, modifications of skipped connection, such as U-Net++ uses nested skip pathway to down-sampling along with encoder. Moreover, R2U-Net utilizes the residual convolutional unit to conduct skipped connection and along with RCNN as the backbone as shown in Fig 2(C). Between the variants, these approaches modify backbones of U-Net or re-design skip pathways or combine both, which are implemented based on the encoder-decoder-based architecture [33–37]. In contrast, the proposed approach adopts novel structure, encoder-parallel decoders architecture, to adapt and implement the individual separation tasks as shown in Fig 2(D).

The proposed semi-Siamese U-Net outputs individual conductivity distributions from EIT image, not just segmentation maps, which is entirely different from other variants of U-Net.

## Multi-weighted loss

Apart from the modification of the structure of the U-Net architecture, the study also modified the loss function such that the separation tasks were associated with each other. The new loss (L_total_), which is called multi-task loss (Eq 1), is expressed as the sum of losses (L_lung_ and L_heart_) in the multiple-separation task. Therefore, we added up the two losses by the individual loss function components so that the semi-Siamese U-Net model can be trained simultaneously on the two separation tasks, i.e., joint learning.

The lung-related impedance changes dominate in the EIT images; therefore, it is difficult to distinguish the cardiac impedance changes. For this reason, we provide weight to the losses to compensate for the difference in conductivities regarding bioelectric properties and to force the network to learn the segmentation mapping of heart rigorously. In other words, the importance of heart imaging during network learning is increased by adding weight on losses.

Hence, the idea of modifying the loss using weights was adopted, in which the weighted losses (W_lung_ and W_heart_) optimize separation performance.

$$L_{total}=w_{lung}\;L_{lung}+w_{heart}\;L_{heart}$$ (1)

Based on the concept of multi-task and weighted losses, attempts have been made on different combinations of weight, which here refers to the hyperparameters; for example, the weight of the cardiac changes was increased and the weight of the lung-related changes was reduced in the following experiments.

## Dataset preparation

### FEM phantom

**Fig 3:**
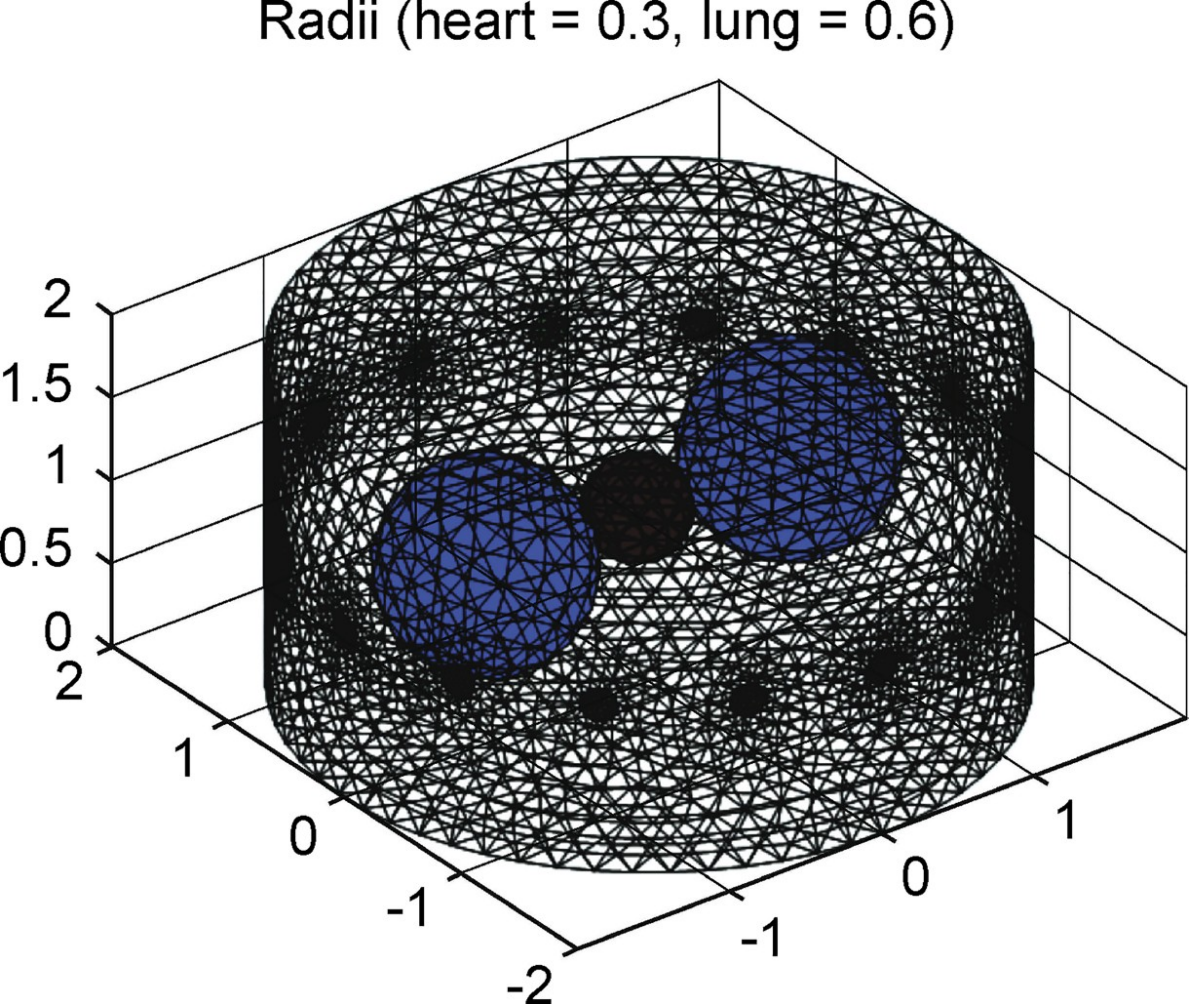
3D EIDORS spheres model.

Fig 3 shows the FEM phantom designed for simulation of thorax, including 16 electrodes and three spheres, which contain the lung region of 0.5 as well as a heart region of 2 of background conductivity. Both radii of the lungs and heart were programmable for simulation of activity in the thorax. For generating the training dataset, the radii of lungs and heart were varied from 0.3–0.6 and 0.1–0.3 (unit: arbitrary), respectively. The FEM phantom and reconstructed images were built and obtained using EIDORS. The FEM phantoms were constructed according to the abovementioned parameters, and image reconstructions were performed to generate the EIT images by solving the forward and inverse problems with EIDORS, as was implemented by Adler et al. in MATLAB [38, 39].

**Fig 4:**
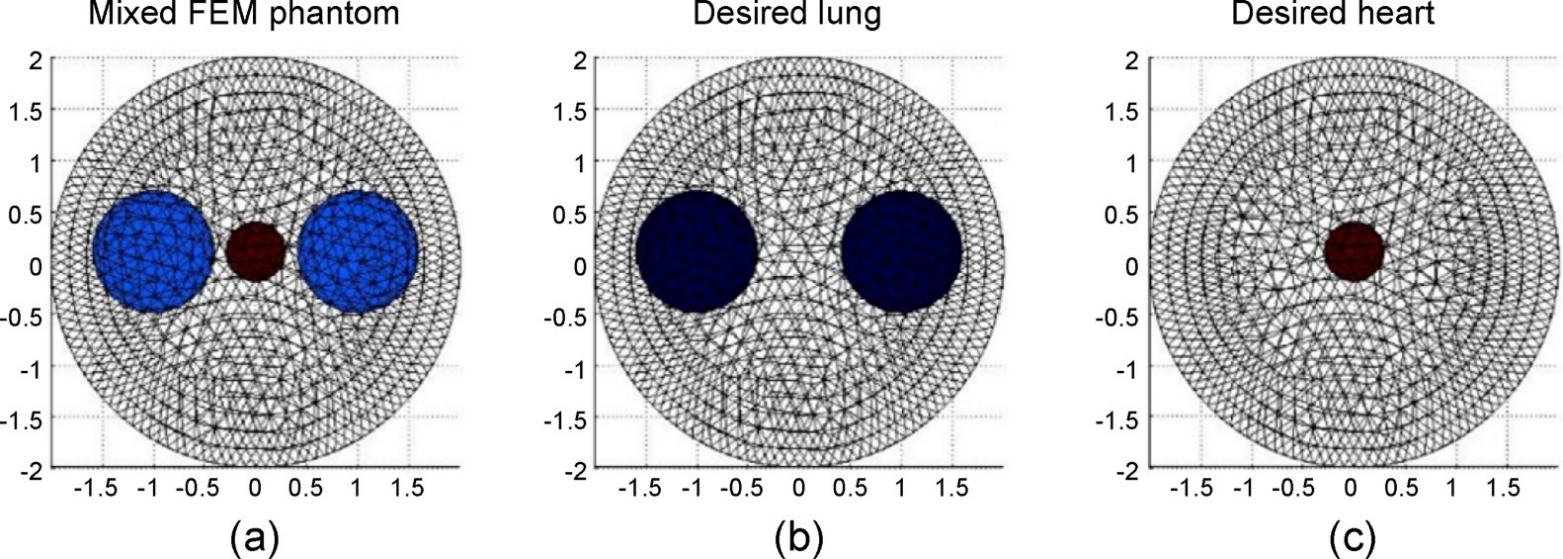
FEM phantoms designed for generating the training data. (a) FEM phantom comprising lungs and heart in the simulation of human thorax. The radii of lung and heart were varied from 0.3–0.6 and 0.1–0.3. (b) The corresponding lung phantom, which is considered as the training target. (c) The corresponding heart phantom, which is considered as the training target.

**Fig 5:**
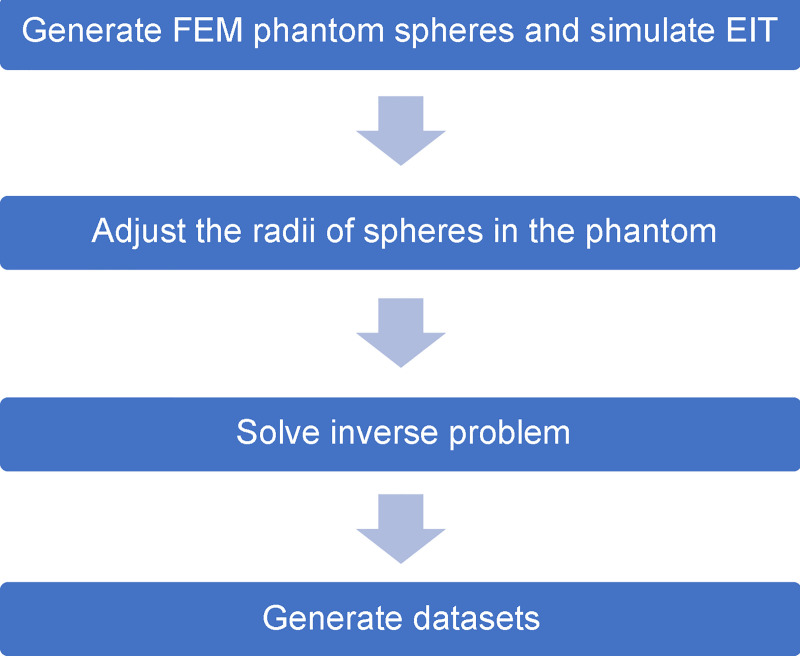
Flowchart of the method used to generate images to train the semi-Siamese U-Net.

The experimental design of the FEM phantom comprises varying combinations of three spheres. The combinations represent the interactions between the contractions and expansions of the heart and lungs as shown in Fig 4. Fig 4(A) represents the simulation of the thorax with both of the lungs and heart, whereas Fig 4(B) and 4(C) represent the lungs and heart in the thorax, respectively. Fig 5 flowchart illustrates the experimental design used for generating FEM phantom and obtaining the reconstructed images for training data.

In order to obtain an efficient dataset for training, up to 3600 EIT inverse problems were solved as shown in Fig 4. Of the 3600 EIT images, the images in the first subset are 1200 original reconstructed images as inputs by solving the inverse problem of the FEM phantom which is the aforementioned design. The second group of 1200 images are the desired outputs segmentation map of the lungs, which are the corresponding reconstructed images, as shown in Fig 4(B). The last 1200 images are corresponding reconstructed images as shown in Fig 4(C), which are the desired outputs segmentation map of the hearts. Each of the images from the three subsets formed a group, out of which two were target images and the other was the corresponding original mixed EIT image.

### Dataset preprocessing

Before training, the dataset was divided into three subsets, 10% of the dataset was considered as a subset to test the trained model. This testing set was an independent set that was not used for the training. The rest of the dataset was apportioned into training 90% and validation 10%.

## Training of semi-Siamese U-Net

The input mixed EIT images and their corresponding desired images were used as segmentation maps to train the semi-Siamese U-Net with the following dependencies and implementation.

### Dependencies

The mentioned semi-Siamese U-Net was implemented with Keras 2.3.1 functional API, which is a minimalist, highly modular neural networks library, written in Python 3.6.8 with TensorFlow-GPU 1.14.0 backend. The following experiments were conducted using a computer with intel core i7-9700K processor (3.60 GHz, 8 MB cache) CPU, 32 GB RAM, and NVIDIA GeForce GTX 1080 Ti GPU.

### Hyperparameters

In this work, the cross-entropy was considered as loss function, and the Adam optimizer was used to minimize the loss, which was used for the original U-Net [25]. In the semi-Siamese U-Net model, we adopted the Adam optimizer with the same parameters as those adopted for the original U-Net. The semi-Siamese U-Net architectural details are described in Table 1.

As mentioned in the proposed architecture, the novel architecture consists of a contracting path and two parallel expanding paths, which added a branch from the end of the contracting path to form expanding path 1 and path 2, to perform the multi-task deep learning for image separation. The symmetric layers are concatenated together to extract spatial information from different layers, such as block1 concatenate block9, block2 concatenate block8, block3 concatenate block7, block4 concatenate block6. The convolutions follow the original U-Net design.

## Experiments

In this study, we first developed the novel semi-Siamese U-Net model and investigated the feasibility of separation tasks in EIT. Thereafter, experiments were conducted to explore the impact of using the multi-task weighted losses on model learning performance. Subsequently, the results of predicted images were examined, in which input image is the pattern corresponding to maximally expanded lungs and contracted heart.

### Baseline model

The proposed architecture, semi-Siamese U-Net, is targeted to and tailor made for separation application in EIT image. Semi-Siamese U-Net is developed based on the state-of-the-art U-Net architecture; therefore, the semi-Siamese U-Net performance was compared with the U-Net architecture as the baseline model.

The number of parameters of the models are listed in Table 2. The proposed semi-Siamese U-Net requires fewer number of parameters than U-Net, and 0.69-times the parameters of a dual U-Net. In EIT separation, the dual U-Net needs twice the number of parameters of two U-Nets to perform two separation tasks.

**Table 2:** The parameter of models in our experiments.

Model	Dual U-Net (original)	Semi-Siamese U-Net (proposed)
Parameters	62,063,370	43,221,322

Although the proposed architecture had a lesser number of parameters, it outperformed the U-Net architecture in separation performance.

### Evaluation metrics

Dice similarity coefficient (DICE), also known as the Sørensen–Dice index, is one of the most commonly used metrics in semantic segmentation, which measures the similarity between two sets of data. DICE for two images A and B is defined as the ratio of twice the area of overlap and the total number of pixels in both images. This metric ranges from 0 to 1, where 0 signifies no overlap, and 1 signifies perfectly overlap between the predicted and ground truth. The DICE score is computed as follows:

$$DICE=\frac{2*area\;of\;overlapped}{total\;area\;of\;A\;and\;B}$$

In this study, the semi-Siamese U-Net has to learn not only the precise location but also the corresponding impedance amplitude. Therefore, another evaluation metric, mean absolute error (MAE), was adopted for evaluating the separation performance. It is the measure of error between the predicted image and ground truth. In our case, the MAE is utilized to compute the pixel-wise error between the predicted and desired images. A low MAE implies high accuracy, i.e., the difference between the predicted and desired images is small. The MAE score is computed as follows:

$$MAE=\frac{\sum \left|\bar{Y}-Y\right|}{N}$$

where $\bar{Y}$ represents the desired image as ground truth, *Y* corresponds to the predicted segmentation image, and N is the number of pixels of an image.

Therefore, by taking the two metrics, dice and MAE, we not only consider precise segmentation but also the difference in impedance level.

## Results

### Performance trade-offs for selection of model for fine tuning

The model was trained for 100 epochs using the aforementioned hyperparameter as in Table 1 with the checkpoints. In each run with 10 epochs, the best results were recorded.

A 30-epochs model was selected to fine tune the training model with weighted loss owing to the fact that after 30 epochs, the lung separation performance greatly increased, but the performance of heart separation was not showing progressive improvement and was in fact getting worse.

Thereafter, attempts were made to progressively modify the weight of losses to investigate the trade-off between the performance and number of training epochs, and the impact of increasing weight on heart loss was explored.

The comparison results of separation of the lung and cardiac related images by the proposed semi-Siamese U-Net model without adjusting weights loss and baseline U-Net model are presented in Table 3. Table 4 compares the predicted results of the semi-Siamese U-Net model with the baseline model by introducing adjusted weighted loss. The different weights of heart loss are presented in Table 4. For better readability, the DICE and MAE values have been converted to percentages in the tables.

**Table 3:** Comparisons of performance between semi-Siamese U-Net and U-Net.

		Semi-Siamese U-Net	U-Net	Relative improvement
DICE (%)	Heart	93.56	88.38	5.18
	Lungs	98.84	96.65	2.19
MAE (%)	Heart	2.67	3.36	0.69
	Lungs	2.67	5.85	3.18

**Table 4:** Results of using different weighted losses for heart in semi-Siamese U-Net and comparison with the baseline model.

	Semi-Siamese U-Net					
	W_heart_ = 1.5		Relative improvement	W_heart_ = 1.8		Relative improvement
DICE (%)	Heart	97.62	9.24	Heart	99.75	11.37
	Lungs	99.54	2.89	Lungs	99.85	3.20
MAE (%)	Heart	0.64	2.72	Heart	0.2	3.16
	Lungs	1.49	4.36	Lungs	0.4	5.54

Superiority of performance of semi-Siamese U-Net over that of U-Net

#### Multi-segmentation task

The evaluation metrics results for both proposed semi-Siamese U-Net and baseline U-Net models in 30 epochs are presented in Table 3.

From Table 3, it can be observed that the performance of the proposed semi-Siamese U-Net model surpasses that of the U-Net architecture in EIT separation tasks. In particular, notable improvement is observed for DICE of heart image segmentation. For heart image separation, the proposed model achieved 5.18% relative improvement in DICE over U-Net. The U-Net scored under 90% DICE, which implies that the separation of the center and high conductivity region is challenging. As lung impedance image separation is relatively easier, the U-Net scored over 96% DICE, while the proposed model achieved 2.19% relative improvement in DICE. Moreover, the semi-Siamese U-Net gave lower MAE in the predicted results of both heart and lungs. Therefore, our proposed model attains superior separation performance over typical U-Net. The relative improvement can be attributed to the interaction between branch structure and multi-task means.

#### Weighted losses

As described in Performance Trade-Offs for Selection of Model for Fine Tuning section, based on the 30-epoch model, we further modified the paraments to investigate the separation performance according to the different weighted losses of heart data. The results of the semi-Siamese U-Net introducing the weighted losses, compared with those of the baseline model, are summarized in Table 4. It is clear that superior separation performance could be achieved by increasing the weighted loss of heart. Both heart- and lung-impedance image separation performances in terms of DICE and MAE were superior to those of the baseline model. Notably, the adding to 1.8 heart weighted loss reached the under 1% of MAE, which is 0.2% and 0.4% for heart and lungs separation results, respectively. Moreover, DICE of heart separation was 99.75%, which was 11.37% relative improvement; meanwhile, lung separation was also more accurate, 99.85% and 3.20% in terms of DICE and relative improvement. This can be attributed to the heart-data weighted loss contribution and joint learning.

#### Number of epochs

**Fig 6:**
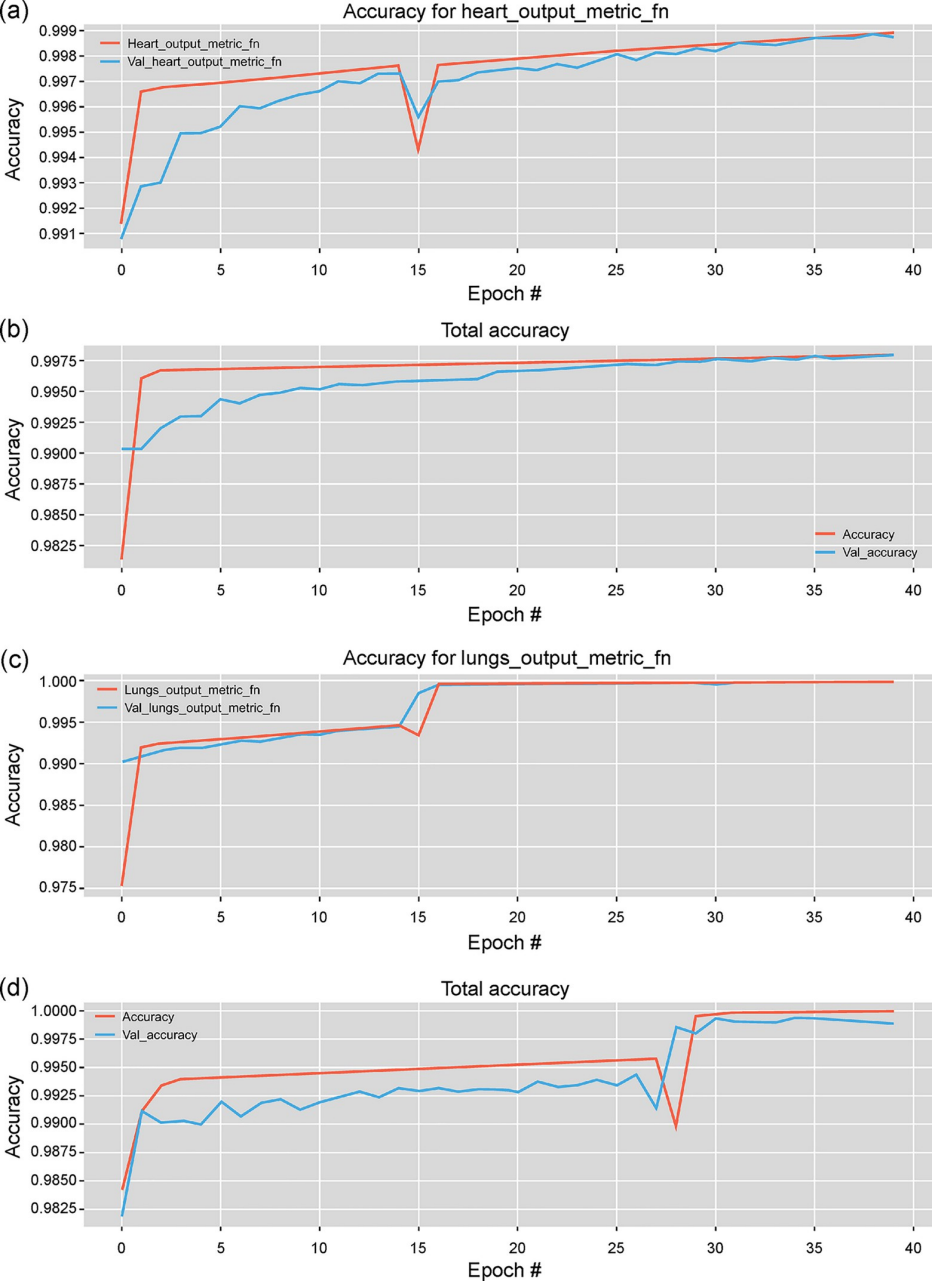
Performance progression according to the number of epochs with training and validation data. Illustration of heart impedance image separation performance with (a) semi-Siamese U-Net and (b) U-Net. Lung impedance image separation performance with (c) semi-Siamese U-Net and (d) U-Net.

In Fig 6, the performances of the models with different number of epochs during training is shown. The results were obtained from the proposed and baseline model with the same parameters. It can be observed that for both heart and lung impedance image separation tasks, the accuracy of the proposed semi-Siamese U-Net model converges rapidly.

For heart-impedance separation, the proposed model achieved 99.7% accuracy at 5 and 11 epochs with the training and validation datasets, respectively, while the baseline model required more than 15 epochs, as shown in Fig 6(A) and 6(B). For lung impedance separation, after around 15 epochs, the accuracy of our model rapidly converged to over 99.8% accuracy, while that of the baseline model converged to the same accuracy after 28 epochs, as shown in Fig 6(C) and 6(D). As indicated by the results, the U-Net architecture could indeed succeed in achieving the separation of EIT images; the proposed model could efficiently learn how to separate the two types of information from mixed reconstructed EIT images, and the learning of lung-impedance separation was relatively easier.

#### Predicted results

**Fig 7:**
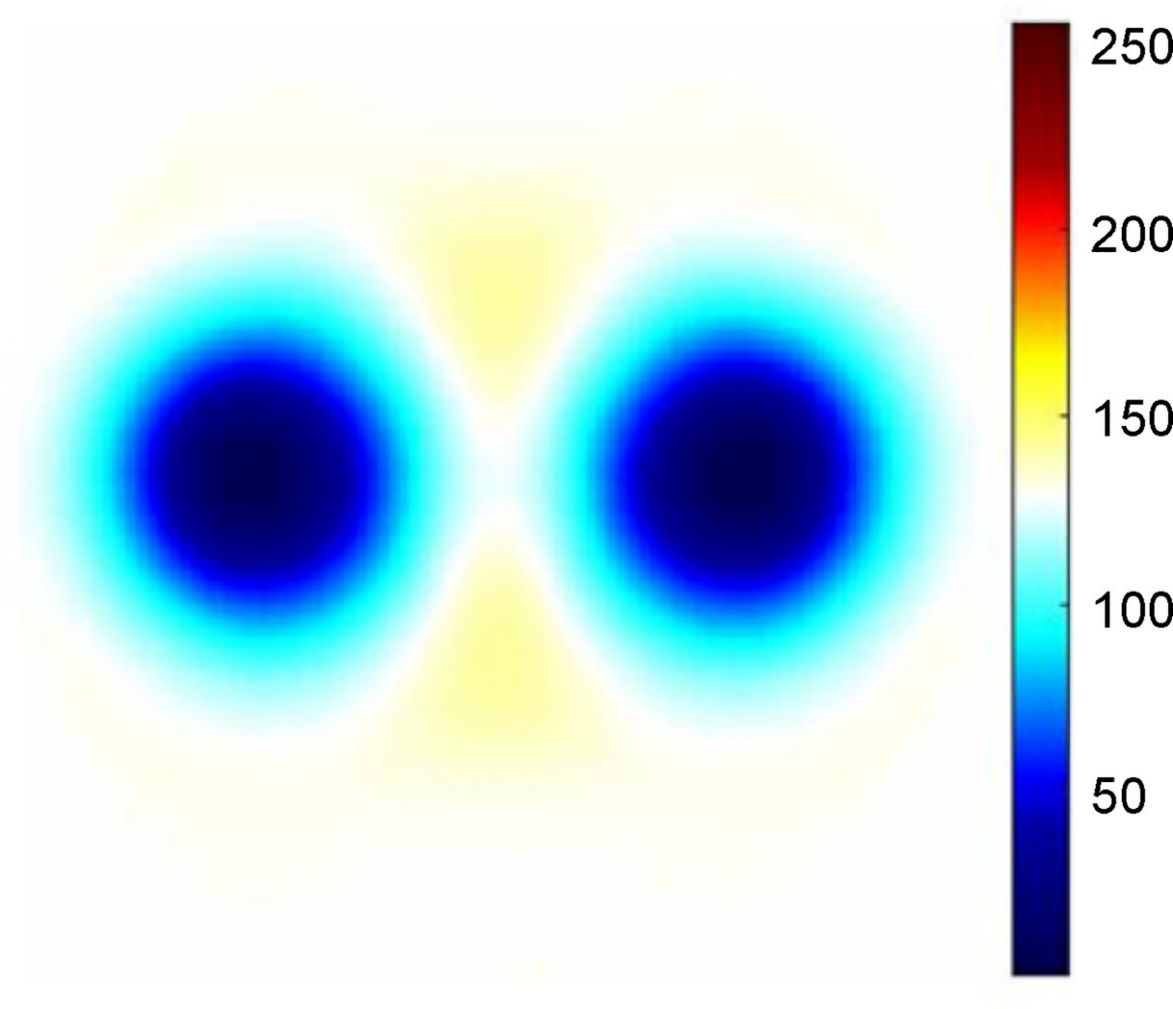
Example of original reconstructed EIT image.

**Fig 8:**
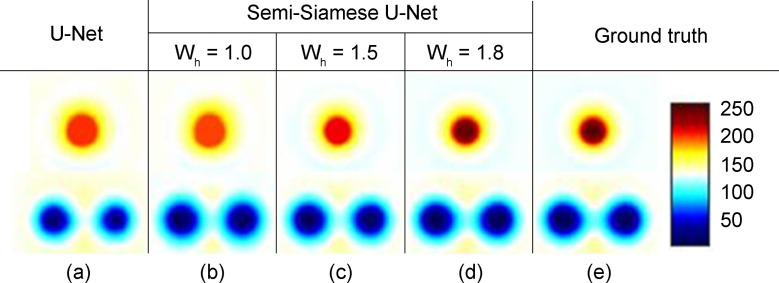
The predicted separation results of heart and lungs.

Figs 7 and 8 show the reconstructed and predicted separated EIT images, respectively. As shown in Fig 7, distinct lungs and suppressed heart images were seen in the EIT reconstrued image. In Fig 8, the images were obtained using semi-Siamese U-Net with increased heart-impedance weighted losses and the baseline model.

The baseline model U-Net demonstrated distinguishable separation results in our experiments as shown in Fig 8(A). The state-of-the-art U-Net architecture has made significant advances in the domain of EIT image separation; however, the proposed semi-Siamese U-Net model takes this a step further and performs better, by attaining 11.37% relative improvement, especially in learning heart-impedance image separation task, as shown in Fig 8(D). As shown by the results, the semi-Siamese U-Net clearly delineates boundaries and approximates the conductivity, which converges accurately.

The U-Net model under-separated the heart image, it only achieved 88.38% DICE, as shown in Fig 8(A). After using the trained semi-Siamese U-Net model, DICE was considerably improved to 93.56%, which implies that the semi-Siamese U-Net architecture more precisely localized the heart region, as shown in Fig 8(B). MAE of predicted lung image relatively improved by 3.18%, which implies that the semi-Siamese U-Net separated the content from the original mixed image more accurately, as shown in Fig 7. Therefore, the predicted results of the proposed semi-Siamese U-Net architecture were superior to those of classical U-Net in not only the predicted heart image but also the lung image.

In Fig 8(B), the boundary of predicted heart image was convergent, and the conductivity of predicted lung image was close to the ground truth. This could be attributed to the branch structure of the semi-Siamese U-Net wherein the parallel paths undergo more specialized learning.

Applying the weights to the losses of heart-impedance greatly reduced MAE to under 1% and increased DICE to over 99%. As shown in Fig 8(B)–8(D), the boundary of the predicted heart image progressively converged, and the conductivity of predicted lung image steadily approached the ground truth. In Fig 8(D), with 1.8 as the value of weighted loss of heart impedance, the semi-Siamese U-Net predicted the images of the heart and lungs with satisfactory accuracy. This could directly be attributed to the impact of weighted loss.

Thus, apart from the metrics, there has been a relatively remarkable improvement and the predicted images of these individual parts are visually more accurate.

## Discussion

Herein, we propose a novel deep-neural-network-based method to separate EIT images based on the U-Net architecture, whose structure was modified to employ shared parameters at contracting layers as well as add a branch at expanding layers, forming parallel expanding paths. Along with the modified structure, the weighted losses synergistically enhanced the image quality of the center area. Distinguishable images were generated from mixed EIT images by the trained semi-Siamese U-Net model with satisfactory accuracy.

In the past, attempts have been made to separate the heart and lung impedance changes in EIT images. Such approaches required a long period of time or prior knowledge, such as ECG or frequency spectrum information, to separate individual parts; therefore, the separated impedance images could not immediately reflect sudden changes in the signals. Moreover, these approaches were based on linear transformation method to decompose EIT images; however, the EIT data are severely nonlinear and ill-posed.

Presently, although the frequency spectrum-based method [13–16] has been employed in commercial EIT systems as a postprocessing method to analyze the mixed EIT images, it is not a reliable clinical solution because obtaining prior information on the threshold setting for each patient is challenging. In addition, there is no gold standard for evaluating the separation results from mixed EIT data.

To address these problems, we proposed a novel nonlinear model and deep supervised learning herein to force the network to rigorously learn segmentation mapping of the heart with respect to the center area, owing to its poor sensitivity for imaging.

As the results show, the trained semi-Siamese U-Net is capable of more accurately separating the heart and lung images from mixed EIT images owing to the tailored modification in structure and multi-weighted loss, as compared with classical U-Net architecture. The branch structure yields more precise localization for separation of the two components; multi-weighted losses not only precisely separate the impedance image but also capture the individual conductivity corresponding to mixed contents. The trained model can achieve end-to-end co-separation without any prior knowledge, such as ECG or frequency spectrum information.

The study is based on the FEM phantom to simulate the thorax EIT for exploring the feasibility and effectiveness of a deep neural network separation method.

A limitation of the current study concerns the FEM model used in this study, which consists of simplistic spheres. In future studies, models with more sophisticated geometries, instead of spheres, will be used to approximate a real human thorax. Thus, the semi-Siamese U-Net model could be generalized well in practice. The trained semi-Siamese U-Net will be further applied to real human EIT data, and the separation effect of the EIT image obtained by the different reconstruction algorithms will be investigated, notably, Shield back-projection algorithm, FEM-based linearized Newton-Raphson algorithm, and Graz consensus reconstruction algorithm (GREIT).

## Conclusions

EIT imaging is a nonlinear and ill-posed problem, and its main drawback is poor spatial resolution; therefore, reconstruction of distinguishable EIT images that include the heart and lung-induced impedance changes is challenging. To address the need for separation of heart- and lung-related bioimpedance images, we proposed the semi-Siamese U-Net architecture. The suggested architecture, based on state-of-the-art U-Net, exploits the advantages of redesigned expanding paths and multi-weighted losses for deep supervision learning of the co-separation tasks. According to the results of the FEM-based experiments, the proposed semi-Siamese U-Net presented was able to achieve co-separation of heart- and lung-induced impedance changes in the EIT image. Hence, this approach successfully captures the heart information using nonlinear end-to-end deep neural networks. To the best of the knowledge of the authors, this is the first study to introduce a state-of-the-art U-Net to address the issue of separation in EIT field and propose the modification and extension of the classical U-Net tailored for EIT application.

In future work, such information could be further useful to derive not only the ventilation image but also cardiac impedance image in which bedside diagnosis would be available, and lung EIT will be of utmost importance in diagnoses, such as ARDS related or respiratory-cardiovascular system related disorders.
